# *FILAMENTOUS FLOWER *controls lateral organ development by acting as both an activator and a repressor

**DOI:** 10.1186/1471-2229-12-176

**Published:** 2012-10-01

**Authors:** Oliver Bonaccorso, Joanne E Lee, Libby Puah, Charles P Scutt, John F Golz

**Affiliations:** 1 Department of Genetics, University of Melbourne, Royal Parade, Parkville, VIC 3010, Australia; 2 Laboratoire de Reproduction et Développement des Plantes, UMR 5667- CNRS/INRA/Université de Lyon, École Normale Supérieure de Lyon, 46, allée d'Italie 69364, Lyon Cedex, 07, France

**Keywords:** YABBYs, *Arabidopsis thaliana*, Leaf patterning and development, Adaxial-abaxial polarity, Lateral organ formation

## Abstract

**Background:**

The YABBY (YAB) family of transcription factors participate in a diverse range of processes that include leaf and floral patterning, organ growth, and the control of shoot apical meristem organisation and activity. How these disparate functions are regulated is not clear, but based on interactions with the LEUNIG-class of co-repressors, it has been proposed that YABs act as transcriptional repressors. In the light of recent work showing that DNA-binding proteins associated with the yeast co-repressor TUP1 can also function as activators, we have examined the transcriptional activity of the YABs.

**Results:**

Of the four *Arabidopsis* YABs tested in yeast, only FILAMENTOUS FLOWER (FIL) activated reporter gene expression. Similar analysis with *Antirrhinum* YABs identified the FIL ortholog GRAMINIFOLIA as an activator. Plant-based transactivation assays not only confirmed the potential of FIL to activate transcription, but also extended this property to the FIL paralog YABBY3 (YAB3). Subsequent transcriptomic analysis of lines expressing a steroid-inducible FIL protein revealed groups of genes that responded either positively or negatively to YAB induction. Included in the positively regulated group of genes were the polarity regulators *KANADI1* (*KAN1*), *AUXIN RESPONSE FACTOR 4* (*ARF4*) and *ASYMMETRIC LEAVES1* (*AS1*). We also show that modifying FIL to function as an obligate repressor causes strong *yab* loss-of-function phenotypes.

**Conclusions:**

Collectively these data show that FIL functions as a transcriptional activator in plants and that this activity is involved in leaf patterning. Interestingly, our study also supports the idea that FIL can act as a repressor, as transcriptomic analysis identified negatively regulated FIL-response genes. To reconcile these observations, we propose that YABs are bifunctional transcription factors that participate in both positive and negative regulation. These findings fit a model of leaf development in which adaxial/abaxial patterning is maintained by a regulatory network consisting of positive feedback loops.

## Background

The YABBY (YAB) family of transcription factors regulates various aspects of vegetative and floral development in flowering plants. First identified in *Arabidopsis*, YABs have a characteristic structure that includes an N-terminal zinc finger domain and a C-terminal YABBY domain containing a helix-loop-helix motif similar to that found in the high mobility group (HMG) of proteins [1,2]. Analysis of the zinc finger domain has shown that it mediates homo- and heterodimerization between the YABs, as well as interactions with other proteins [3,4]. In contrast, the YABBY domain is associated with non-specific DNA-binding [5]. Phylogenetic analysis distinguishes five sub-families of YABs in the angiosperms, represented by the FILAMENTOUS FLOWER/YABBY3 (FIL/YAB3), YAB2, YAB5, CRABS CLAW (CRC) and INNER NO OUTER (INO) clades. In eudicots such as *Arabidopsis*, the so-called vegetative YABs - *FIL*, *YAB3*, *YAB2* and *YAB5* - are expressed in the abaxial domain of developing leaf and floral organ primordia [2,6], whereas *CRC* is restricted to the developing carpel and nectaries, and *INO* is expressed specifically in the outer integument of the ovule [7,8]. Characterisation of the vegetative YABs through mutant and gain-of-function analyses has shown that they regulate cell identity in developing organs and thus play an important role in establishing organ polarity and subsequent lamina growth [1,2,4,6,9]. This regulation is complex, however, as loss of FIL and YAB3 activities is associated with the partial adaxialisation of organs [2], whereas combined loss of FIL, YAB3 and YAB5 results in organ abaxialisation [4]. Similar observations have been made in *Antirrhinum*, where mutations in the FIL/YAB3 ortholog, GRAM, are associated with a loss of abaxial cell identity, whereas when combined with mutations in the YAB5 ortholog, PROLONGATA (PROL), they result in a loss of adaxial cell identity [10]. YABs fit into a highly complex and redundant network of transcription factors and small RNAs that promote organ polarity (for example see [11]). Factors that promote adaxial identity include the AS2, ARP and the class III HOMEODOMAIN-LEUCINE ZIPPER (C3 HD-ZIP) transcription factors, as well as the trans-acting small interfering RNAs generated by the miR390-TAS3-RDR6 pathway [12-14]. In contrast, the KANADI (KAN1, KAN2 and KAN3) and AUXIN RESPONSE FACTOR (ETTIN (ETT)/ARF3 and ARF4) classes of transcription factors promote abaxial identity, together with the microRNAs *miR165*/*166*[15-18]. The precise position of YABs within these networks is not certain, but based on *yab* loss-of-function phenotypes it has been proposed that YABs integrate adaxial-abaxial patterning with a program of lamina growth [6].

In addition to promoting lamina growth, vegetative YABs also prevent shoot apical meristem (SAM) regulators from being expressed in the developing leaf primordia. In the absence of vegetative YAB activity, *KNOX* and *WUSCHEL* (*WUS*) expression is detected in leaves, where it is associated with the formation of SAM-like structures [6,19]. YABs also play a significant role in regulating SAM activity, as the inflorescence meristem of *fil* mutants is noticeably enlarged and displays aberrant phyllotaxy [20,21]. In addition, these meristem defects are associated with laterally expanded expression of the meristem regulators *WUS* and *CLAVATA3* (*CLV3*) [20]. Major disruptions in SAM size and organisation, culminating in SAM arrest, are also observed in higher order *Arabidopsis* and *Antirrhinum yab* mutants [4,6,10]. This aspect of YAB function is apparently conserved in angiosperms as a recent study has shown that *TONGARI-BOUSHI1*, a *FIL*-like *YAB* from rice, controls floret meristem activity [22]. As YAB accumulation in both *Arabidopsis* and *Antirrhinum* is limited to the abaxial domain of developing lateral organs and floral primordia [20,23], their SAM-promoting activity presumably reflects non-cell-autonomous activity [10,20].

Recent studies have begun to address how YABs function at a molecular level. For instance, physical and genetic interactions between the vegetative YABs and the co-repressors LEUNIG (LUG) and the closely related LEUNIG_HOMOLOG (LUH) suggest that YABs act in repressive complexes [4]. LUG and LUH share structural and functional similarity with TUP1, Groucho (Gro) and TLE co-repressors that are present in yeast, *Drosophila* and vertebrates [24]. Given this similarity, it is likely that LUG and LUH display a similar range of interactions with DNA-binding factors as that described for fungal and animal co-repressors. In this regard it is interesting to note that in yeast, some DNA-binding proteins are capable of functioning as activators when not associated with a repressor complex [25,26]. Several recent studies have also identified plant transcription factors with dual activities, behaving as either activators or repressors on different sets of target genes [27-29] or in different tissues [30]. This raises the intriguing possibility that some DNA-binding proteins associated with Gro-like co-repressors may also possess bifunctional activity. In this study, we test this possibility by examining the regulatory properties of the vegetative YABs. We show, using a combination of transient transactivation assays and genome-wide transcriptomic analysis, that FIL and possibly YAB3 function as activators during vegetative development. Consistent with the biological importance of activation activity, fusing the repressive motif SRDX to FIL results in a dominant negative phenotype when expressed in plants. Based on transcriptomic analysis, several polarity regulators were identified as FIL targets. Significantly, two of these target genes, *KAN1* and *ARF4* are thought to lie upstream of the YABs. We therefore propose a model in which YAB proteins maintain leaf polarity by establishing a positively reinforcing feedback loop following the emergence of adaxial/abaxial patterning.

## Results

### FIL and FIL-like YABs function as activators in yeast

**Figure 1:**
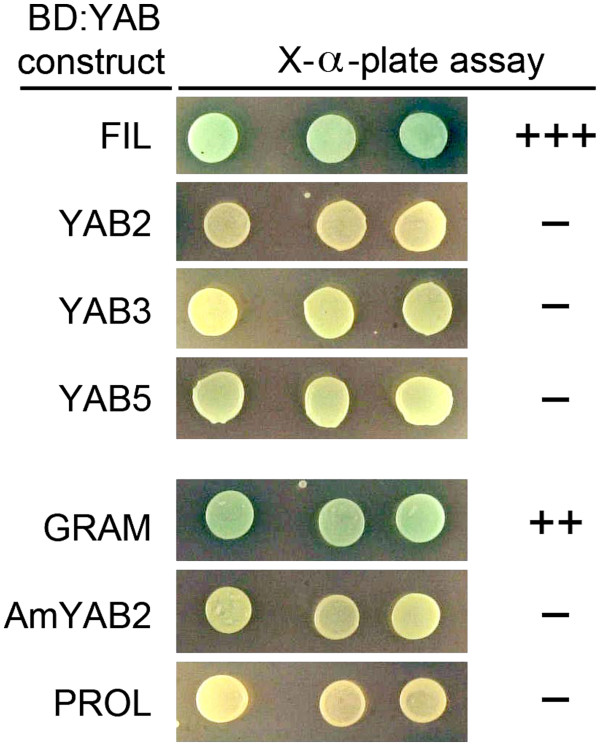
**YAB transcriptional activity in yeast. **Three independently transformed yeast strains expressing *Arabidopsis *YABs (FIL, YAB2, YAB3, YAB5) or *Antirrhinum *YABs (GRAM, AmYAB2, PROL) fused to the GAL4 DNA-binding domain (BD) were assayed for MEL1 reporter activity using an X-α-gal plate assay. Colour change after 4 h (+++), after 16 h (++), or no colour change after 24 h (−) are shown.

We previously used the yeast two-hybrid assay to examine physical interactions between the YABs and various components of the LUG co-repressor complex. In testing YAB constructs for autoactivation, we discovered that yeast expressing the GAL4 DNA binding domain (BD) fused to FIL produced a noticeable colour change after 4 h growth on media containing X-α-Gal ([4]; Figure 1). Interestingly, this property is not shared with the closely related YAB3 or with other vegetatively expressed YABs (YAB2 and YAB5) (Figure 1). To determine whether activation is a conserved feature of the FIL subgroup of YABs, we examined whether the *Antirrhinum* FIL ortholog, GRAM, behaves as an activator in yeast. For comparison, we also tested the YAB5 ortholog PROL and the YAB2 ortholog AmYAB2. When grown on media containing X-α-Gal, yeast lines expressing BD:GRAM produced a colour change after 16 h, whereas no colour change was detected with either BD:PROL or BD:AmYAB2. While the behaviour of YAB proteins in yeast does not necessarily reflect their activity in the plant, these results nonetheless raised the intriguing possibility that the FIL subgroup of YABs function as activators.

### *In planta* assays indicate that FIL/YAB3 can function as transcriptional activators

**Figure 2:**
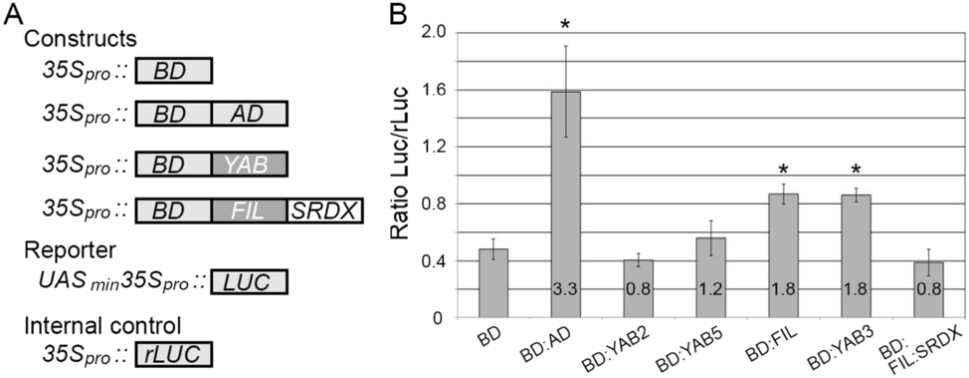
**Transcriptional activities of vegetatively expressed YAB proteins. **(**A**) Outline of constructs used for transactivation assays. AD, GAL4 activation domain; BD, GAL4-DNA binding domain; YAB, vegetatively expressed YABs (FIL, YAB2, YAB3, YAB5); SRDX, repressive domain (see text for details); UAS, BD binding site; LUC, Firefly Luciferase; rLUC, *Renilla *Luciferase. (**B**) YAB transcriptional activation assays with Gal4 BD and BD-AD used as negative and positive control respectively. rLuc was used as an internal control to determine the relative bioluminescence for each sample (ratio Luc/rLuc). Numbers in the shaded boxes indicate fold activation as calculated by dividing total Luc activity of samples by the baseline values arising from the *35S*_*pro*_*::BD *construct. Asterisks indicate significant differences (Student's *t*-test; p < 0.05) and error bars indicate SEM.

To further investigate the properties of the *Arabidopsis* YABs, we used an *in vivo* plant transcription assay to determine whether the vegetatively expressed YABs (FIL, YAB2, YAB3, YAB5) function as activators. YABs were translationally fused to GAL4 BD (Figure 2A) and introduced into *Arabidopsis* leaves along with the luciferase reporter *UAS::LUC*. Following transfection, proteins were extracted from leaf tissue and assayed for luciferase activity. We first established a baseline level of luciferase activity by assaying extracts from leaves transfected with *35S*_*pro*_*::BD*, and then found that a construct expressing BD fused to the Gal4 activation domain (AD; *35S::BD:AD*) produced a 3.3 fold increase in luciferase activity. While BD:YAB2 and BD:YAB5 constructs produced no more than baseline luciferase activity, BD:FIL and BD:YAB3 both activated the *UAS::LUC* reporter, resulting in a 1.8-fold increase in luciferase activity (Figure 2B). These results not only confirm that FIL functions as an activator in plants, but extend this function to the closely related YAB3 protein.

### Genome-wide transcript profiling following FIL activation

**Figure 3:**
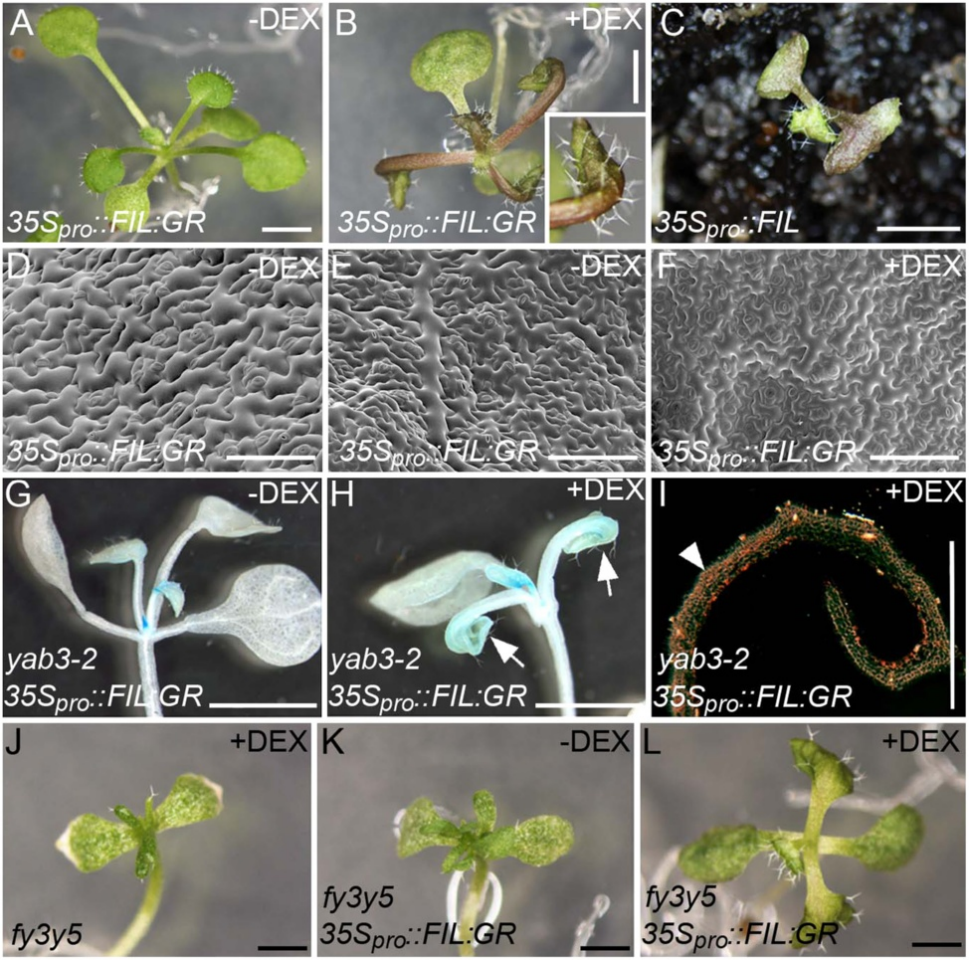
**Vegetative phenotypes associated with steroid-induced constitutive activation of FIL. **(**A**,**B**) A *35S*_*pro*_*::FIL:GR *plant grown on media without DEX (**A**) or with DEX (**B**). Inset shows close-up view of an epinastic leaf. (**C**) *35S*_*pro*_*::FIL *plants displaying an intermediate phenotype. (**D**-**F**) Scanning electron micrographs showing the adaxial (**D**, **F**) or abaxial (**E**) surface of mature leaves of *35S*_*pro*_*::FIL:GR *plants grown on media without DEX (**D**,**E**) or with DEX (**F**). (**G**,**H**) Histochemical staining for YAB3:GUS activity in *yab3-2*/*35S*_*pro*_*::FIL:GR *plants grown in the absence of DEX (**G**) or with DEX (**H**). Arrows indicate prolonged GUS activity in the first true leaves to emerge following germination. (**I**) Section through a histochemically stained leaf shown in (**H**) viewed by dark field optics. Arrowheads indicate adaxial accumulation of GUS activity. (**J**-**L**) Twenty one day-old *fil yab3 yab5 *(abbreviated as *fy3y5*) triple mutants (**J**) and *fy3y5*/*35S*_*pro*_*::FIL:GR *(**K**,**L**) lines grown on media with DEX (**J**, **L**) or without DEX (**K**) under short days. Scale bar: 1 mm in (**A**-**C**,**G**,**H**, **J**-**L**), 100 μm in (**D**-**F**, **I**).

To identify genes that are positively regulated by FIL during shoot development, we opted to assay genome-wide changes in the *Arabidopsis* transcriptome following rapid induction of FIL activity. Lines expressing an inducible form of FIL (*35S*_*pro*_*::FIL:GR*) were generated by fusing FIL to the ligand-binding domain of the rat glucocorticoid receptor (GR; [31]). In the absence of the synthetic hormone dexamethasone (DEX), these lines were phenotypically normal, but when grown on media with DEX, or sprayed with DEX, they produced downward curling epinastic leaves that accumulated high levels of anthocyanin (Figure 3A,B). These characteristics are similar to those reported for *35S*_*pro*_*::FIL* lines displaying an intermediate over-expression phenotype (Figure 3C; [2]). Moreover, induction of FIL activity was also associated with leaf abaxialisation, as the adaxial epidermal pavement cells of DEX-treated *35S*_*pro*_*::FIL:GR* leaves had an abaxial morphology, appearing smaller and more irregularly shaped than cells of mock treated leaves (Figure 3D-F). Abaxialisation was subsequently confirmed when GUS activity derived from the *yab3-2* allele, a gene trap that produces abaxially-restricted GUS activity [19], was detected in the adaxial domain of leaves of DEX-grown *yab3-2/35S*_*pro*_*::FIL:GR* plants (compare Figure 3G-I). Activation of FIL:GR also completely suppressed the formation of narrow or needle-like leaves of the *yab* triple mutants when activated in this background (Figure 3J-L).

Having shown that the FIL:GR fusion is biologically indistinguishable from FIL, we assessed transcriptional changes in the shoots of ten-day-old plants following activation of FIL:GR. As plants were grown under short-day conditions, shoot tissue consisted of mature cotyledons, hypocotyl, and approximately four fully emerged leaves at various stages of development. These included leaves undergoing leaf blade expansion (first true leaves), as well as leaves that had only just emerged from the shoot. Although not visible, initiating organ primordia were also part of these samples. RNA was extracted from this tissue following exposure to DEX or a mock treatment, and used to probe Affymetrix ATH1 genome microarrays. Each treatment consisted of four biological replicates with expression being sampled at 4 h and 8 h post-DEX induction. Following normalization of the expression data and comparisons between mock and DEX treated samples, differentially expressed genes were ranked according to their fold change in expression. We considered a gene differentially expressed if there was a 1.5 fold or greater change in expression following DEX treatment, and if this expression difference was associated with a 0.05 or smaller *q*-value (see Additional file 1 for a list of differentially expressed genes). The 4 h dataset included 252 up-regulated genes and 251 down-regulated genes, while the 8 h dataset had 166 up-regulated and 184 down-regulated genes. Of these, 219 genes (107 up-regulated/112 down-regulated) were present in both datasets and thus represent a group of genes that consistently respond to FIL activation over an 8 h time period (Group A; Additional file 2). A second group of genes were differentially expressed at 8 h but were not present in the 4 h dataset (Group B; Additional file 2). Because Group A represents genes with robust and consistent responses to FIL activation, we primarily focused on this group.

The close match between the number of group A genes showing reduced expression and the number showing elevated expression following DEX treatment suggested that FIL functions as both an activator and a repressor. To determine whether these two functions are associated with different biological processes, we used gene ontology analysis to identify terms that are enriched in the up- and down-regulated gene sets. Down-regulated genes were enriched for GO terms associated with growth processes (corrected *p*-value = 0.088) and responses to auxin (corrected *p*-value = 0.01), whereas up-regulated genes were enriched for terms associated with metabolic processes (auxin biosynthesis; *p*-value = 0.0466), responses to stimuli including abiotic stress (osmotic), biotic agents (fungal, bacterial) and chemicals (ethylene, chitin), as well as multi-cellular organismal processes (p = 0.0864) involved in lateral root formation. Based on this analysis, it appears likely that FIL regulates distinct processes by functioning as an activator in some cases and as a repressor in others.

Previous work has established that YABs promote organ polarity, and ectopic *FIL* expression is associated with the partial abaxialisation of leaves (this study; [1,2,4,6]). Consistent with this function, we found that two well-defined abaxial-promoting regulators, *KAN1* and *ARF4*, are elevated ~2-fold following FIL:GR activation (Table 1; Additional file 1). Surprisingly, *ETT*/*ARF3*, a gene that functions redundantly with *ARF4* in promoting abaxial cell fate [18], and other *KAN* family members associated with abaxial patterning (*KAN2*, *KAN3*[32,33]) were not found in either Group A or B. This suggests that FIL activates some, but not all cell polarity regulators.

**Table 1:** **Group A FIL-response genesselected for further analysis**

**Identifiers**		**Fold change detected by microarray**		**Ratio**	**Fold change detected byqRT-PCR**
**AGI**	**Name and Symbol**	**4h**^**a**^	**8h**^**a**^	**8h/4h**	**4h**^**a,b**^
AT5G04340	C_2_H_2_ zinc-finger transcription factor (CZF2)	2.72	13.05	4.80	1.96
AT2G38470	WRKY family transcription factor (WRKY33)	5.61	15.11	2.69	3.09
AT1G56650	MYB75/PAP1	9.78	22.20	2.27	3.35
AT1G06160	Ethylene response factor (ERF59)	8.66	19.05	2.20	8.79
AT3G23550	MATE efflux family protein	14.24	20.02	1.41	22.73
AT5G60450	AUXIN RESPONSE FACTOR 4 (ARF4)	3.64	4.14	1.14	2.74
AT4G36410	Ubiquitin-conjugating enzyme 17 (UBC17)	4.11	4.69	1.14	3.13
AT5G16560	KANADI1 (KAN1)	2.96	3.35	1.13	3.33
AT3G45640	Mitogen-activating protein kinase 3 (MPK3)	2.55	2.80	1.10	2.24
AT3G62150	ATP-BINDING CASSETTE B21	5.35	5.45	1.02	3.15
AT2G28350	AUXIN RESPONSE FACTOR 10 (ARF10)	1.86	1.89	1.02	2.12
AT1G56010	NAC1	3.57	3.16	0.88	1.08^c^
AT5G22580	Stress responsive A/B Barrel Domain	3.44	2.81	0.82	2.5
AT1G55200	Protein kinase	4.13	3.26	0.79	2.28
AT3G13672	Seven in absentia-like protein	4.06	2.94	0.72	3.97
AT3G13690	Protein kinase	3.17	2.09	0.66	2.30
AT5G07580	Ethylene response factor (ERF5)	3.87	2.29	0.59	2.93
AT2G37630	ASYMMETRIC LEAVES1 (AS1)	-^d^	1.70	-	2.01
AT3G15570	NON-PHOTOTROPIC3 (NPH3)	−1.89	−3.49	1.84	−1.80
AT3G23050	IAA7	−1.74	−2.04	1.17	−1.82
AT2G40610	Expansin (Exp8)	−3.28	−3.76	1.15	−2.97
AT5G05690	Cytochrome P450 (CPD)	−2.21	−1.69	0.76	−1.50
AT1G20190	Expansin (Exp11)	−6.17	−4.57	0.74	−3.71

^a^Length of DEX treatment. ^b^Statistically significant fold change (*p*<0.05) was calculated using a Student's *t*-test; ^c^FC is not statistically significant, ^d^Group B gene.

### Validation of microarray data

**Figure 4:**
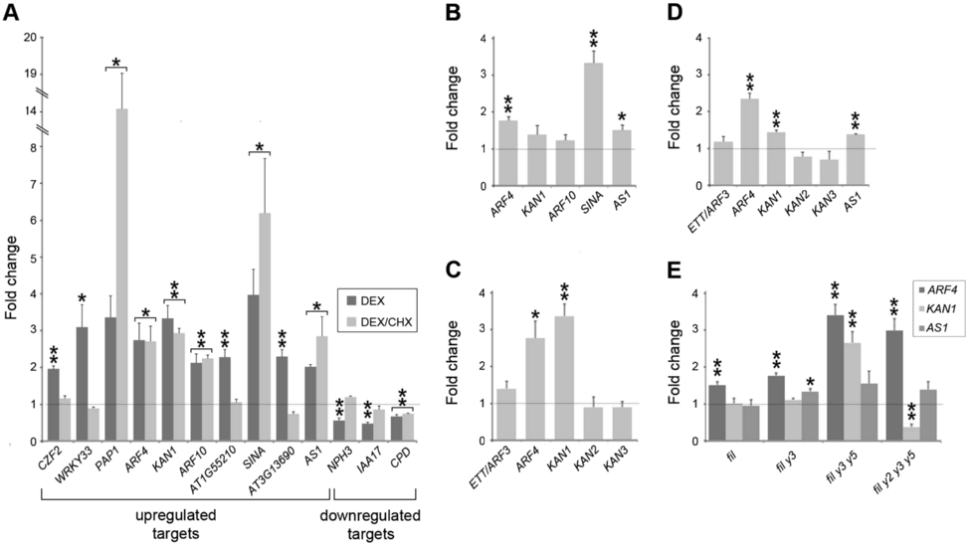
**FIL-response genes that areimmediate targets of FIL.** (**A**) Fold change in expression of FIL-response genes in ten-day-old *35S*_*pro*_*::FIL:GR* seedlings following a 4 h DEX or DEX/CHX treatment. Brackets indicate genes that display significant transcriptional responses to both treatments and hence mark direct targets of FIL. (**B**) Response of selected positively regulated FIL-response genes in 10 day-old *FIL*_*pro*_*::FIL:GR* seedlings following a 4 h DEX treatment. (**C**,**D**) Induction of abaxial polarity regulators in 10 day-old *35S*_*pro*_*::FIL:GR* (**C**) or *35S*_*pro*_*::YAB3:GR* (**D**) seedlings following a 4 h DEX treatment. (**E**) Expression of *KAN1*, *ARF4* and *AS1* in *fil* single and *yab* double, triple and quadruple mutants. Expression in a minimum of three biological replicates was determined using quantitative real-time RT-PCR and normalized first to a housekeeping gene and then to mock treatment controls. Asterisks mark significant differences determined by a Student’s *t*-test (one asterisk, 0.01<p<0.05; two asterisks, 0.001<p<0.005) and error bars are SEM. The grey line marks the expression level expected if there is no response to treatment.

To verify our microarray data, expression of selected Group A genes in *35S*_*pro*_*::FIL:GR* shoot tissue exposed to DEX or a mock DEX treatment for 4 h was assessed by quantitative RT-PCR. Included in this group were genes associated with polarity regulation (*KAN1*, *ARF4*, *AS1*) as well as genes associated with auxin (*ARF10*, *IAA7*, *NPH3*), ethylene responses (*ERF5*, *ERF59*) and anthocyanin regulation (*MYB75*/*PAP1*). With the exception of *NAC1*, all of the genes displayed a statistically significant change in expression following DEX application, which corroborated the microarray data (Table 1, Figure 4A). As microarray analysis was performed 4 h after DEX treatment, it is likely that many of the differentially expressed genes are direct downstream targets of FIL. This was tested by examining responsiveness of selected FIL-target genes to DEX induction in the presence of the translational inhibitor cycloheximide (CHX). Using an inducible transactivation system, where GUS activity is controlled by a DEX-inducible synthetic transcription factor GR-LhG4 (abbreviated to *35S*_*pro*_^*I*^*> > GUS*; [34]), we showed that combined exposure to CHX and DEX was sufficient to suppress GUS activity (see Additional file 3). Having established the effectiveness of CHX treatments, we next examined the transcriptional responses of twelve Group A genes (nine in the up-regulated class and three in the down-regulated class) and one up-regulated Group B gene following combined DEX/CHX exposure. Seven of these genes showed a statistically significant change in expression following these treatments and were thus considered direct FIL targets (Figure 4A).

A complicating factor in our analysis is that *FIL:GR* expression is not confined to the abaxial domain, and thus transcriptional changes induced by FIL activation may not reflect the normal behaviour of the endogenous protein. To address this, we generated a *FIL*_*pro*_*::FIL:GR* line in which inducible FIL activity is limited to the abaxial domain of developing leaves. Growing this line on media with DEX did not result in a YAB over-expression phenotype, although some leaf epinasty was apparent (see Additional file 4). We next used qRT-PCR to assess the transcriptional response of five positively regulated FIL-response genes in the *FIL*_*pro*_*::FIL:GR* line following a 4 h DEX treatment. All five genes responded positively to induction, although statistically significant elevation was only apparent for three of these: *ARF4*, *SINA* and *AS1* (Figure 4B). Despite the limited nature of this survey, the observed similarity in transcriptional response between the *35S*_*pro*_*::FIL:GR* and *FIL*_*pro*_*::FIL:GR* lines suggests that many of the FIL-response genes identified in our study are indeed immediate targets of FIL.

To confirm that members of the *KAN* family (*KAN2*, *KAN3*) and the *ARF4*-related gene *ETT*/*ARF3* did not respond to FIL activation, we used qRT-PCR to assay their expression in the *35S*_*pro*_*::FIL:GR* line following a 4 h DEX treatment. Consistent with the microarray experiment, this analysis failed to detect significant changes in the expression of these genes (Figure 4C). We next considered whether the FIL paralog YAB3 regulated *ARF4*, *KAN1* and *AS1*. To test this possibility, we generated an inducible *YAB3* line (*35S*_*pro*_*::YAB3:GR*) and showed that growth on media containing DEX induced a YAB over-expression phenotype (see Additional file 4). qRT-PCR revealed significant elevation of *ARF4*, *KAN1* and *AS1* following DEX treatments, but not of *ETT*/*ARF3*, *KAN2* or *KAN3* (Figure 4D). This analysis not only indicates that FIL and YAB3 regulate the same target genes, which is consistent with the observed redundancy between these close paralogs [2], but that there is also a high degree of specificity in this regulation, as related family members do not respond to YAB induction.

### Analysis of FIL-regulated polarity regulators in mutant lines

Our data indicated that FIL, and possibly other vegetative YABs, regulate *ARF4*, *KAN1* and *AS1* during vegetative development. To test this possibility further, we analysed the expression of these genes in shoot tissue derived from 28-day-old *fil* mutants, as well as from mutant combinations containing a progressively reduced complement of active vegetative *YAB* genes (*fil yab3*, *fil yab3 yab5* and *fil yab2 yab3 yab5* mutants). A complicating factor in this analysis is the observed abaxialisation of *yab* triple and quadruple mutants [4], which would be expected to cause elevated expression of abaxial polarity regulators. Consistent with this, we found that both *ARF4* and *KAN1* expression was elevated in triple *yab* mutants, with *ARF4* expression remaining high in the quadruple mutants (Figure 4E). Unlike *ARF4*, *KAN1* expression was significantly reduced in quadruple mutants, suggesting that *KAN1* is regulated directly and positively by the YABs. *AS1* expression remained unchanged in the multiple *yab* mutant lines (Figure 4E), with the exception of *fil yab3* mutants, in which it showed a slight increase. While this analysis clearly demonstrates the predominant role of *YABs* in regulating *KAN1*, regulation of *ARF4* and *AS1* presumably involves other factors that function redundantly with the YABs.

Our expression studies suggested the possibility that ectopic expression of the polarity regulators *KAN1* and *ARF4* following FIL:GR activation may be the cause of the polarity defects observed in the *35S*_*pro*_*::FIL:GR* line when continuously exposed to DEX. To test this, *kan1* and *arf4* mutations were introduced sequentially or together into the *35S*_*pro*_*::FIL:GR* background and their phenotype assessed following continuous exposure to DEX. Lines receiving DEX treatment displayed the same leaf epinastic and adaxialisation phenotype as that of *35S*_*pro*_*::FIL:GR* lines (see Additional file 5). This suggests that the abaxialisation phenotype associated with constitutive YAB activity is the result of multiple factors being ectopically expressed during leaf development.

### Expression of *FIL:SRDX* induces *yab* loss-of-function phenotypes

The ERF-associated amphiphilic repressor motif (EAR/SRDX; [35]) has been used to convert positively-acting transcription factors into dominant repressors. Thus, to address the importance of transcriptional activation for YAB function, we investigated the effects of constitutive *FIL:SRDX* expression in plants. Before performing these experiments, we confirmed that the SRDX motif suppressed FIL activation by showing that a FIL:SRDX fusion was incapable of activating the luciferase reporter in plant-based transactivation assays (Figure 2B).

**Figure 5:**
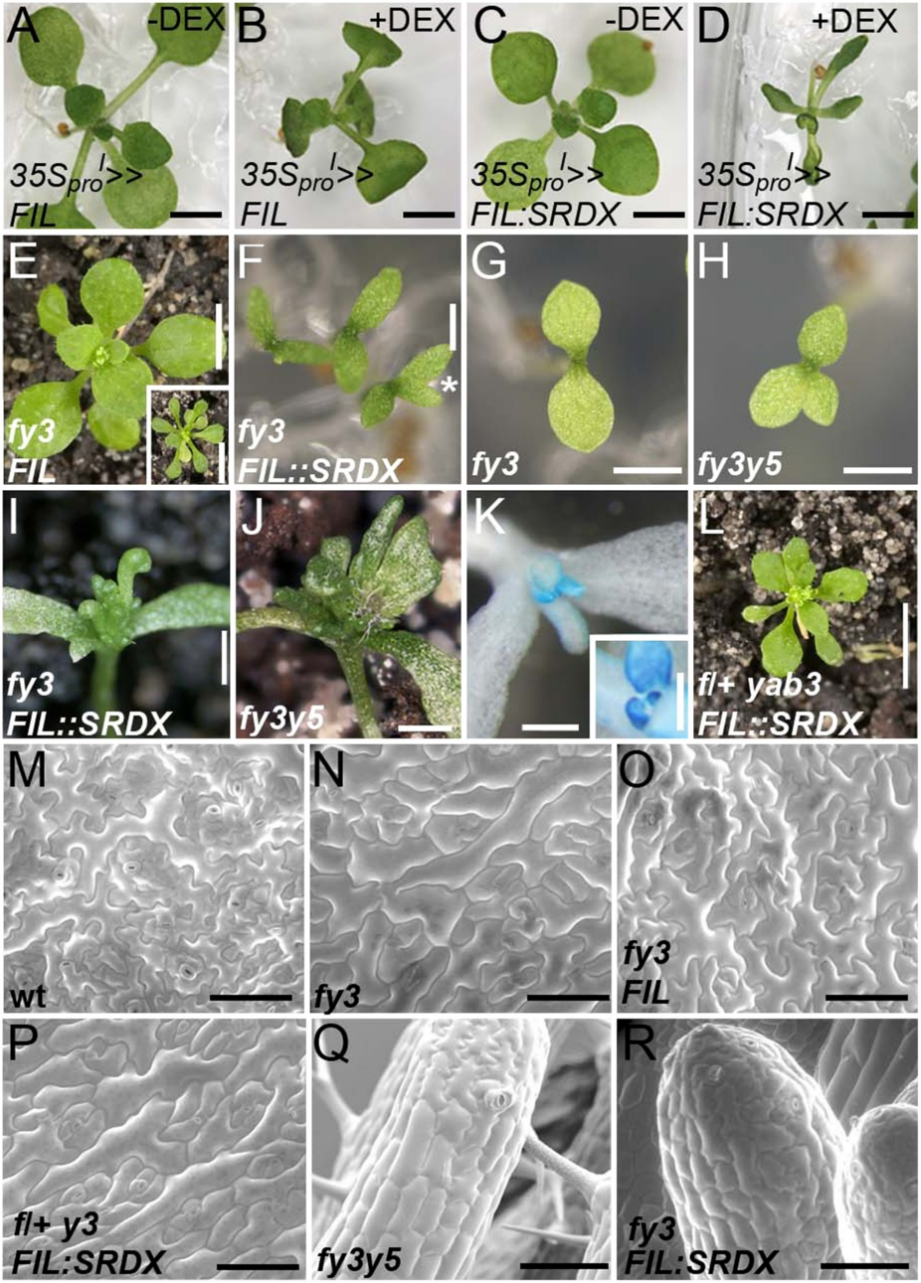
**Phenotypes induced by adominant negative *FIL:SRDX *construct.** (**A**,**B**) *35S*_*pro*_^*I*^*>>FIL* plants grown on media without DEX (**A**) or with DEX (**B**). (**C**,**D**) *35S*_*pro*_^*I*^*>>FIL:SRDX* plants grown on media without DEX (**C**) or with DEX (**D**). (**E**) A *fil yab3*/*FIL*_*pro*_*::FIL* plant displaying full complementation. Inset: *fil yab3* double mutant plant. (**F**) *fil yab3*/*FIL*_*pro*_*::FIL:SRDX* seedlings showing narrow cotyledons that are sometimes bifurcated (asterisk). Cotyledons of *fil yab3* (**G**) and *fil yab3 yab5* mutant seedlings (**H**). (**I**) *fil yab3*/*FIL*_*pro*_*::FIL:SRDX* plant with needle-like leaves. (**J**) *fil yab3 yab5* triple mutant plant with narrow and needle-like leaves. (**K**) Histochemical staining for YAB3:GUS activity in a *fil yab3*/*FIL*_*pro*_*::FIL:SRDX* plant. *YAB3* promoter activity is detected throughout young radial leaves. Inset: *fil yab3*/*FIL*_*pro*_*::FIL* stained for GUS activity. (**L**) A *fil*/+ *yab3*/*FIL*_*pro*_*::FIL:SRDX* plant with a *fil yab3* mutant leaf phenotype. (**M**-**R**) Scanning electron micrograph showing the abaxial epidermis of wildtype (**M**), *fil yab3* (**N**), *fil yab3 FIL*_*pro*_*::FIL* (**O**) and *fil*/+ *yab3*/*FIL*_*pro*_*::FIL:SRDX* (**P**) leaves. Note that the larger cell morphology in (**N**,**P**) is due to leaf adaxialisation. (**Q**,**R**) SEM showing epidermal cell morphology of *fil yab3 yab5* needle leaves (**Q**) and those of *fil yab3*/*FIL*_*pro*_*::FIL:SRDX* plants (**R**). Scale bars are 5 mm in (**E**,**L**) and inset in (**A**); 2 mm for (**A**-**D**, **F**-**H**); 1 mm for (**I**,**J**); 200 μm for (**K**) and inset in (**K**); 100 μm in (**M**-**R**).

To compare the effects of constitutive FIL:SRDX expression to that of FIL, plants were transformed with either a *35S*_*pro*_*::FIL:SRDX* or *35S*_*pro*_*::FIL* construct. While fifty-five *35S*_*pro*_*::FIL* primary transformants were recovered following Basta treatment, the same selection yielded only a single *35S*_*pro*_*::FIL:SRDX* transformant. This plant had a wild type appearance, suggesting that the *FIL:SRDX* transgene was unlikely to be active in this line. Given the failure to isolate significant numbers of transgenic plants, we assumed that embryonic or early seedling activity of the *FIL:SRDX* transgene was likely to be associated with lethality. To circumvent this problem, we used the GR:LhG4/pOP transactivation system to drive post-embryonic expression of *FIL* transgenes (*35S*_*pro*_*::GR:LhG4*/p*OP::FIL;* abbreviated to *35S*_*pro*_^*I*^*> > FIL*). Growth of *35S*_*pro*_^*I*^*> > FIL* lines in the presence of DEX produced the same epinastic cotyledon phenotype as *35S::FIL:GR* lines exposed to DEX (Figure 5A,B compare with Figure 3B), although subsequent leaf development exhibited weaker phenotypes, even when the DEX concentration was doubled (data not shown). In contrast, the early stage of *35S*_*pro*_^*I*^*> > FIL:SRDX* growth on media with DEX was characterised by the development of narrow, upwardly curling, hyponastic cotyledons and leaves (Figure 5C,D). The fact that this phenotype differs from the one associated with constitutive *FIL* expression is consistent with FIL:SRDX no longer functioning as an activator.

In contrast to previous experiments using SRDX fusions [36,37], the presence of this motif was not sufficient to induce a strong loss-of-function phenotype in *35S*_*pro*_^*I*^*> > FIL:SRDX* lines. This could be due to levels of FIL:SRDX not being sufficiently high to interfere with the activity of the native FIL/YAB3 proteins. To address this, we introduced a *FIL*_*pro*_*::FIL:SRDX* construct into *yab3* mutants that were heterozygous for the *fil* mutation (*fil*/+ *yab3*) and analysed the phenotype of transgenic *fil yab3* mutants in the subsequent generation. As a control for these experiments, we first showed that *FIL*_*pro*_*::FIL* complemented the *fil yab3* mutant phenotype (n = 19; Figure 5E). None of the *FIL*_*pro*_*::FIL:SRDX* lines complemented the *fil yab3* phenotype, instead, the majority (n = 19/30) had cotyledons that were noticeably smaller than those of *fil yab3* mutants and, like *fil yab3 yab5*, displayed frequent bifurcations (Figure 5F-H). Leaves arising in over half of the *fil yab3*/*FIL*_*pro*_*::FIL:SRDX* lines (n = 13/19) were similar in appearance to *fil yab3 yab5* mutant leaves in being radial and extremely short (Figure 5I, J; [4]). Presence of YAB3:GUS activity throughout the developing leaves of these lines indicated that these organs were partially or fully abaxialised (Figure 5K). In lines where this abaxialisation phenotype was most prevalent (n = 6/13), transgenic *fil*/+ *yab3* sibs also had a *fil yab3* leaf phenotype (Figure 5L), which is consistent with FIL:SRDX acting dominantly to suppress native FIL activity.

Analysis of leaf epidermal cell morphology confirmed that *FIL*_*pro*_*::FIL* complemented the polarity defects of *fil yab3* mutants, as abaxial cells displayed a wildtype morphology (Figure 5M-O). In contrast, *fil*/+ *yab3*/*FIL*_*pro*_*::FIL:SRDX* cells were larger and less irregular than wildtype abaxial cells and similar in appearance to the partially adaxialised cells of *fil yab3* mutants (Figure 5N,P). Indeed, SEM analysis of *fil yab3*/*FIL*_*pro*_*::FIL:SRDX* needle-like leaves revealed a mixture of oblong and jigsaw-shaped cells; exactly the same cellular morphology observed in *fil yab3 yab5* triple mutant leaves (Figure 5Q,R; [4]). In summary, these experiments show that inverting the transcriptional activity of FIL through fusions to SRDX leads to a strong *yab* loss-of-function phenotype when expressed in plants lacking native FIL/YAB3 activity. A plausible explanation for this phenotype is that the FIL:SRDX protein functions dominantly by preventing YAB2 and YAB5 from activating their targets. This experiment therefore provides the first direct evidence that YABs function as activators during the early stages of leaf development.

## Discussion

Based on earlier work showing physical and genetic interactions with members of the plant Gro-like family of transcriptional co-repressors, we proposed that YABs function as transcriptional repressors [4,23]. However, genetic analysis indicates that YABs perform their functions independently of these co-repressors. For instance, the conspicuous loss of adaxial cell identity characteristic of *yab* triple or quadruple mutants is not seen in lines lacking co-repressor activity, nor when single or double *yab* mutants are combined with mutations in the *LUG* and *LUH* co-repressors [10,23]. In this study, we investigated the regulatory activity of the vegetative YABs and found that, in yeast-based transcriptional and in plant transactivational assays, FIL functions as an activator. This property is likely shared with the closely related YAB3 protein, but seemingly not with YAB2 and YAB5. Analysis of the ovule specific YAB, INO, indicates that this factor also functions as an activator, although in this case only autoregulation was investigated [8,38]. This conclusion was based on the observation that abundance of *INO* transcripts and activity of an *INO* promoter:reporter construct are reduced in strong *ino* mutants.

Direct evidence for FIL functioning as an activator also comes from our microarray analysis, where changes in gene expression were assessed 4 h and 8 h following DEX-induced activation. Of the response genes identified, approximately half were under positive regulation, whereas the remainder were under negative regulation. Subsequent qRT-PCR analysis incorporating CHX treatments confirmed that half of the genes tested were indeed likely to be direct FIL-targets. An increasingly common way to establish whether a transcription factor functions as an activator is to convert it into a repressor through a fusion to the SRDX motif, and then show that this chimeric protein produces a dominant negative phenotype when expressed *in planta*. While FIL:SRDX expression in wildtype plants did not generate *yab* loss-of-function leaf phenotypes, prominent leaf patterning and growth defects were observed in a *fil yab3* mutant background. This suggests that native FIL and YAB3 proteins suppress the repressive activity of FIL:SRDX, perhaps through competition for target binding sites, as FIL and YAB3 are both expressed at high levels in the abaxial domain of developing leaves. In the absence of competing FIL/YAB3, FIL:SRDX constitutively represses genes normally activated by the remaining vegetative YABs. This implies that all four vegetative YABs function as activators during the early stages of leaf development. While this is consistent with the observed functional redundancy between members of the YAB family [2,4,6], our analysis did not reveal activation activity associated with either YAB2 or YAB5 (Figures 1 and 2). This may reflect the relative insensitivity of our yeast and transactivation assays or, alternatively, a requirement for YAB-interacting proteins that function as activators.

**Figure 6:**
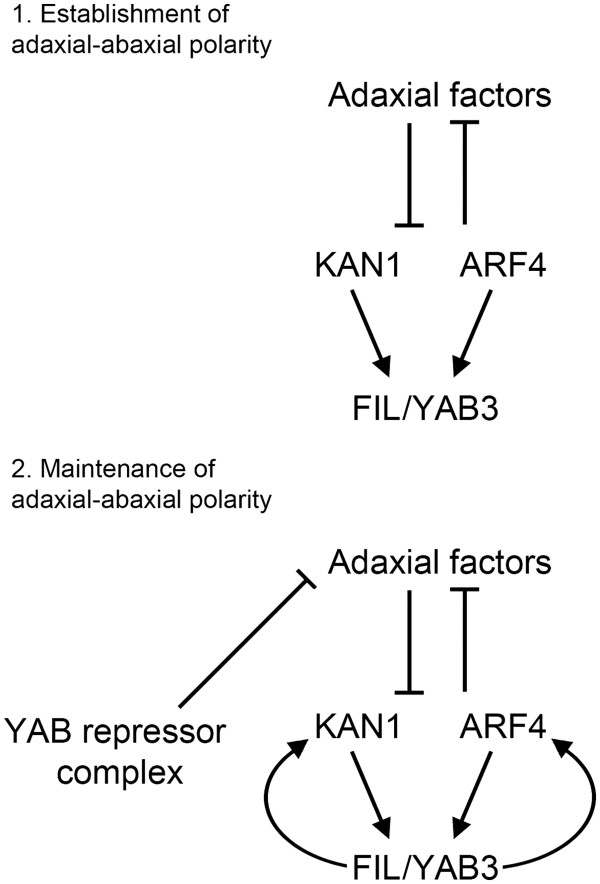
**Model for YAB functionduring the early stagesof leaf development.** Adaxial-abaxial patterning is established during the early stages of leaf development and is closely associated with the onset of *FIL*/*YAB3* expression. FIL/YAB3 maintain *KAN1* and *ARF4* expression through direct positive regulation, which in turn establishes a positive feedback loop. As well as acting as positive regulators, YABs associate with transcriptional co-repressors, forming a repressive complex that potentially targets adaxial-promoting factors. It is likely that this regulatory network is confined to the early stages of leaf development, as at later stages *FIL*/*YAB3* expression is present at high levels in the margins of the growing lamina, but *KAN1* expression is not readily detectable.

Transcription factors that act as both activators and repressors have been reported in animals [39] and several examples are also known in plants. For instance, the meristem-regulator WUSCHEL activates *AGAMOUS* (*AG*) expression in the floral meristem [40], but represses the A-type *Arabidopsis Response Regulator7* (*ARR7*) in the shoot apical meristem [41]. Extensive functional characterisation has confirmed the dual nature of WUS, as well as showing that this activity applies to many more genes [29,42]. Bifunctional activity is also a feature of the senescence-related WRKY53 factor, which acts as an activator or repressor according to sequences surrounding the WRKY-binding motif (W-box) present in the promoters of target genes [27]. On the basis of our results, we propose a model in which YABs are bifunctional, acting either as activators or, when associated with LUG and LUH, as repressors (Figure 6).

A novel aspect of our study is the finding that the abaxial polarity regulators *KAN1* and *ARF4* are likely to be immediate FIL targets. This is supported by the observed increase in *KAN1* and *ARF4* expression in *35S*_*pro*_*::FIL:GR* plants following combined DEX/CHX treatment. Furthermore, finding reduced *KAN1* expression in *yab* quadruple mutants indicates that *KAN1* regulation is a feature common to the other vegetative YABs. In contrast to *KAN1*, *ARF4* expression was elevated in all *yab* mutant backgrounds examined. This finding was surprising, given that FIL acts positively on *ARF4*. Previous studies of *yab* mutants have established that in addition to leaf patterning defects, these lines lack stipules, display retarded growth and have defects in meristem activity [4,6]. *yab* mutants also display defects in auxin-regulated processes, such as vascular patterning and formation of the leaf margin, and have reduced activity of the auxin-responsive reporter *DR5*[6]. Given this, it is possible that increased *ARF4* expression observed in *yab* mutants is a consequence of pleiotropy, and that this obscures the role played by YABs in regulating *ARF4*.

The observation that FIL regulates *KAN1* and *ARF4* is also surprising given that previous studies have placed the YABs downstream of such regulators. For instance, *FIL* expression occurs after the establishment of adaxial-abaxial patterning in developing lateral organ primordia [43], which itself depends on antagonistic interactions between the C3 *HD-ZIPs* and *KAN* genes [15,32]. Thus, *KAN* activity is likely to precede that of the YABs, a hierarchy that is further supported by the observed reduction of *FIL*/*YAB3* expression in developing leaves of *kan* double and triple mutants [15,33]. Similarly, *ARF4* and the related *ETT/ARF3* gene are thought to function upstream of the YABs [44]. Our findings can be reconciled with YABs functioning downstream of these abaxial regulators if it is assumed that FIL functions after the establishment of adaxial-abaxial patterning. Such a scenario is attractive, as activating *KAN1* and *ARF4* following organ patterning would generate a positive feedback loop that maintains abaxial identity (see Figure 6). An important test of this model is to determine whether expression of *KAN*s and C3 *HD-ZIP*s is initially polarized in the organ primordia of *yab* quadruple mutants. This could be addressed using live cell imaging to monitor the dynamics of *KAN* and C3 *HD-ZIP* expression during the early stages of lateral organ development. Another aspect of our model is the role played by the LUG-YAB regulatory complex in the early patterning of the leaf. While our microarray analysis did not identify any known adaxial regulators that are negatively regulated by FIL, previous studies have shown that *lug* mutants enhance the adaxialisation phenotype of *fil yab3* mutant leaves [4]. We therefore propose that the LUG-YAB regulatory complex is involved in the repression of adaxial regulators, although whether this is mediated by direct or indirect regulation remains to be determined. What is currently missing from this model is an understanding of the temporal-spatial distribution of each of these transcriptional activities. For instance, can repression and activation occur in the same cell, or are they spatially separated activities? Determining the expression pattern of positively and negatively regulated YAB-target genes within the developing leaf may help resolve these issues. Additionally, establishing the precise distribution of the LUG-YAB complex within cells of the developing leaf will also provide insight into the likely transcriptional activities of vegetative YABs. While technically challenging, this could be achieved using *in planta* BRET measurements [45].

The activation of *KAN1*, *ARF4* and *AS1* by FIL and YAB3 has recently been integrated into a computational-derived model of sepal patterning [11]. With the exception of *AS1*, the inclusion of these regulatory relationships produced a coherent model with other known patterning pathways. The inconsistency with *AS1* regulation concerns the apparent restriction of *AS1* expression to the adaxial domain of the developing sepal where FIL presumably does not act. However, closer inspection of *AS1* expression domain in sepals revealed that this overlapped with *FIL* during the early stages of organ formation. Thus, FIL regulation of *AS1* could also be incorporated into this model, if it is assumed that there are also adaxial factors that promote *AS1* expression and abaxial factors that repress *AS1* expression during the later stages of sepal development [11].

Auxin distribution is affected in *yab* loss-of-function mutants, leading to disruptions in the marginal region of developing leaves, as well as the formation of multiple cotyledons during embryogenesis [6]. How YABs regulate auxin distribution in the developing leaf blade is not well understood, but may reflect roles in auxin biosynthesis, movement or signalling. Our study confirms the link between the YABs and auxin responses, as many of the genes responding to FIL activation have GO terms associated with either auxin responses or biosynthesis. In addition we show that *ARF4* and *ARF10* are positively regulated by FIL and may thus influence the sensitivity of leaf cells to auxin. It is, however, unlikely that the auxin-associated defects of *yab* mutants are due to reduced activity of *ARF4*, as *ARF4* expression was elevated in triple and quadruple *yab* mutants, presumably as a result of abaxialisation that is associated with the loss of vegetative YAB activity. Instead, we propose that YABs mediate auxin signalling either through direct association with ARFs, or more likely as part of a regulatory complex. Supporting this possibility is evidence that some ARFs interact with components of the LUG regulatory complex ([46] and our unpublished data) and hence may be indirectly associated with the YABs that are also part of these complexes.

## Conclusion

This study has shown that FIL is a bifunctional transcription factor that acts either as an activator or as a repressor. Our finding that the conversion of FIL into an obligate repressor resulted in *yab* loss-of-function phenotypes when expressed in plants provides direct evidence that activation is required for leaf patterning. Consistent with this finding, FIL is likely to function as a direct activator of the abaxial-promoting factors *KAN1* and *ARF4*, as well is adaxial regulator *AS1*. On the basis of these results, it is proposed that FIL generates positive feedback loops that maintain adaxial-abaxial polarity following initial polarization of the leaf primordium. This study has therefore provided important new insights into YAB function, as well as laying the foundations for future studies aimed at understanding these enigmatic transcription factors.

## Methods

### Plant material and growth conditions

*Arabidopsis thaliana* wildtype plants were either Columbia (Col) or Landsberg *erecta* (L*er*). All transgenic plants were generated using an *Agrobacterium* floral dipping method [47] and transgenic lines identified by BASTA treatment. The steroid inducible *FIL* construct *35S*_*pro*_*::FIL:GR*, *35S*_*pro*_*::FIL* and *35S*_*pro*_*::FIL:SRDX* were introduced into Col plants. p*OP::FIL* and p*OP::FIL:SRDX* constructs were introduced into the inducible *35S*_*pro*_*::GR:LhG4* driver line [34] that was kindly provided by Miltos Tsiantis (University of Oxford, UK). *FIL*_*pro*_*::FIL* or *FIL*_*pro*_*::FIL:SRDX* were introduced into *yab3-2* mutant plants segregating for *fil-8,* and transgenic *fil-8 yab3-2* plants were identified by PCR genotyping in either the T1 population or T2 populations. As a result of segregation, *fil-8* was absent in some transgenic T1 plants. These lines were backcrossed to *fil-8 yab3-2* double mutants and *fil-8 yab3-2* progeny identified in subsequent populations.

The steroid-inducible FIL transgene was introgressed into various *yab* mutant backgrounds, which have been described previously and are in either a L*er* or mixed L*er*/Col *er* background [4,19]. To minimize the effects of *er* and background variation, we characterized a minimum of 10 F2 or F3 plants that were wildtype for *ERECTA*. Presence of *yab* mutant alleles and the transgene was confirmed by PCR genotyping. The *35S*_*pro*_*::FIL:GR* transgenic line was also crossed to *kan1-12* and *arf4-3* mutants [16,48] that were kindly provided by Scott Poethig (University of Pennsylvania). Homozygous mutant F2 lines were identified by PCR genotyping. Details of all PCR genotyping are available upon request.

Plants were either grown on soil or on 0.5x Murashige and Skoog media, in a growth room at 18°C or growth cabinet kept at 21°C, under lights for 8 h (short days) or 16 h (long days). For DEX induction, plants were grown on media containing 10 μM DEX/0.1% DMSO or media containing 0.1% DMSO for mock treatment. Alternatively, DEX-induction was performed on soil grown plants. In this case, DEX treatment involved spraying plants with a 20 μM DEX solution containing 0.2% ethanol and 0.05% Silwet L-77 every 2–3 days, or with a solution of 0.2% ethanol and 0.05% Silwet L-77 for mock treatments. In addition to spraying, plants receiving a DEX treatment were watered with a 20 μM DEX/0.2% ethanol solution.

### Constructs

To generate the *35S*_*pro*_*::FIL:GR* construct, the *FIL* coding sequence was PCR-amplified from wildtype inflorescence cDNA using FIL-F3 and FIL-R1.1 primers (see Additional file 6) and a high fidelity Taq polymerase (Kod HiFi; Novagen). Presence of *Xba*I and *Bam*HI sites in these primers allowed the product to be cloned into the equivalent sites of pBIΔGR, a binary vector containing the ligand-binding domain of the rat glucocorticoid receptor (GR) downstream of the cauliflower mosaic virus *35S* promoter (kindly provided by Robert Sablowski, John Innes Centre). As a result, the GR domain was fused in frame with the *FIL* C-terminus. *35S*_*pro*_*::FIL* was generated by amplifying the *FIL* coding sequence with primers cFIL-KpnI and Fil R-X (see Additional file 6) and cloning this product into the *Sma*I and *Xba*I sites located between the *35S* promoter and 3’ UTR of the octopine synthase gene of vector pART7 [49]. *35S*_*pro*_*::FIL:SRDX* was generated by amplifying *FIL* coding sequence with cFIL-KpnI and FILR1.1 primers and cloning the product into *Kpn*I and *Bam*HI sites of BJ36-SRDX, a shuttle vector containing the 12 amino acid synthetic ERF-associated amphiphilic repression motif (SRDX; [35]). This placed the SRDX domain in frame with the C-terminal end of the *FIL* coding sequence. The FIL:SRDX fusion was then moved into the *Kpn*I and *Xba*I sites of pART7. The *FIL*_*pro*_*::FIL* construct was made by placing the 3.8 kb *FIL* promoter [50], amplified from Col genomic DNA with pFIL-Xho and pFIL-KpnI (see Additional file 6), into *Xho*I and *Kpn*I sites upstream of the *FIL* ORF in the shuttle vector BJ36. A similar approach was also used to generate the *FIL*_*pro*_*::FIL:SRDX* construct.

*FIL*-containing cassettes from pART7 and BJ36 were excised with *Not*I and introduced into the *Not*I site of binary vector pMLBART [49]. Binaries were then introduced into *Agrobacterium tumefaciens* (GV3101) by electroporation.

### Yeast assays

The coding sequences of *GRAM, PROL* and *AmYAB2* were amplified from *Antirrhinum majus* vegetative cDNA using a high fidelity Taq polymerase and primers that incorporate *Eco*RI sites (see Additional file 6). The *YAB* sequences were then cloned into the *Eco*RI site of the pGBK-T7 vector (Clontech), resulting in the coding sequence being downstream of and in frame with the Gal4 DNA-binding motif (BD). Constructs were transferred into yeast and transcriptional activity of the chimeric BD-YAB protein was assessed using X-α-Gal plate-based colorimetric assays according to published protocols.

### *In vivo* plant transactivation assays

To generate Gal4 BD fusions, the coding sequences of *FIL*, *FIL:SRDX*, *YAB2*, *YAB3* and *YAB5* were amplified with oligonucleotides listed in Additional file 6 using a high fidelity Taq. PCR products were inserted into the *Sma*I site of vector pMN6 [51], downstream of the BD and *35S* promoter. This resulted in BD being fused in frame to an alanine linker (AAAARS) that was incorporated into the N-terminus of the *YAB* coding sequence by PCR. For the transactivation experiments, pMN6 alone was used as a negative control, while a vector containing both the GAL4 activation (AD) and DNA-binding domains (AD-BD) driven by the *35S* promoter (pMN7; [51]) was used as a positive control. A vector containing the *Renilla* luciferase (rLUC) gene under the control of the *35S* promoter (pRLC) was used as the internal standard and the transactivation reporter was pGLL, a vector with the firefly LUC gene downstream of multiple Gal4 UAS fused to a minimal promoter [51]. Four micrograms of a mixture of BD-YAB construct and firefly LUC reporter (1:1 mass ratio) together with 0.5 μg internal standard were introduced into the abaxial side of mature *Arabidopsis* leaves using particle bombardment according to the manufacturer’s recommendation (BioRad). Bombarded tissue was left for 24 h on 0.5x MS plates under lights before extracting protein into 1x luciferase cell culture lysis reagent (Promega) with proteinase inhibitor (Roche). Activity of LUC in 20 μl of extract was then assessed following the addition of 80 μl assay buffer containing 1x luciferase cell culture reagent, 1x proteinase inhibitor, 0.1 mM EDTA, 20 mM Tricine, 27 mM MgSO_4_, 33 mM DTT, 270 μM CoA, 530 μM ATP and 546 nM luciferin. In a separate assay, rLUC activity was measured following the addition of coelenterazine (Promega) in a standard phosphate buffer. Luminometric assays were performed on three bombardments and were repeated three times using a Mithras LB940 Multimode Microplate Reader.

### Microarray analysis

For experiments involving DEX induction, four pools of ten-day-old *35S*_*pro*_*::FIL:GR* seedlings (grown under short days) were sprayed with a solution containing 10 μM DEX, 0.1% ethanol and 0.015% Silwet L77 (DEX treatment) or a solution containing 0.1% ethanol, 0.015% Silwet L77 (mock treatment), prior to collection at 4 h or 8 h. RNA was extracted from pools of seedlings using a Plant RNeasy Mini Kit (Qiagen). RNA integrity was confirmed by agarose gel electrophoresis before probe synthesis using 300 ng of total RNA per sample according to the AtGenExpress protocol [52]. Affymetrix ATH1 genome arrays were then hybridized and washed using a Genechip fluidics station, and scanned using a Genechip Scanner 3000. Scanned data were processed using the GeneSpring GX software package (Agilent). CEL files were analysed using the GC-Robust Multi Array average method, which incorporates background subtraction, normalization and probe summation. An unpaired Student’s *t* test with a Benjamin-Hochberg false discovery rate correction was applied to the data to obtain *q*-values (corrected *p*-values based on a False Discovery Rate of *p* = 0.05).

### Quantitative real time RT-PCR

For DEX induction, four independent pools of ten-day-old transgenic plants received either a mock or a DEX treatment as outlined above. For DEX/CHX treatments, transgenic plants were first sprayed with a 10 μg/μl cycloheximide solution in 0.015% Silwet L77 and left for 1 h before spraying again with the CHX solution or with a solution containing both 10 μg/μl cycloheximide and 10 μM DEX. Plants were harvested 4 h following these treatments and RNA extracted using an RNeasy kit (Qiagen). For analysis of *yab* mutants, pools of 28-day-old (short-day grown) wildtype (L*er*) plants and *yab* mutants were collected and RNA extracted. Total RNA was first treated with DNase (Ambion) before synthesizing cDNA using an oligo (dT) primer with Superscript III reverse-transcriptase (Invitrogen). PCR reactions were performed in the presence of SYBR-green on a Rotor-Gene 3200 Real-Time Cycler (Corbett Research) using *ACTIN2* (*ACT2*; AT3g18780), *TUBULIN7* (*TUB7*; At2g29550) or *TRANSLATIONALLY CONTROLLED TUMOR PROTEIN* (*TCTP*; At3g16640) as housekeeping controls. Expression levels were first normalized to the housekeeping controls then to the mock treatment, in the case of DEX or DEX/CHX treatments, or to wildtype plants in the case of *yab* mutants. Oligonucleotides used for qRT-PCR are listed in Additional file 6.

### Microscopy

Imaging of the leaf surface was performed on an environmental scanning electron microscope (FEI Quanta) fitted with a Peltier cold stage operating at 2°C. Leaf samples were attached to stubs using carbon adhesive tape before viewing under an accelerating voltage of 12.5Kv and 5.3 Torr vacuum. GUS staining was performed by briefly fixing tissue in 90% acetone and then incubating tissue overnight in a 50 mM phosphate buffer containing X-Gluc and a mixture of potassium ferricyanide and ferrocyanide at 37°C. Tissue was either examined as whole mounts in 70% ethanol or embedded in Paraplast Plus, before being sectioned at 8 μm and viewed under dark-field optics.

### Bioinformatics

The accession code, E-MEXP-3726, may be used to access the microarray data from the ArrayExpress website (http://www.ebi.ac.uk/arrayexpress/)*.*

## Competing interests

The authors declare that they have no competing interests.

## Authors’ contributions

OB conducted the microarray analyses and, together with JEL, quantified expression of FIL-response genes by qRT-PCR. LP made the yeast constructs and together with JEL performed yeast assays looking at the transcriptional activity of *Antirrhinum* and *Arabidopsis* YABs. CPS supervised the plant based transactivation and DEX/CHX qRT-PCR assays and drafted sections of the manuscript. JFG generated all transgenic plants, supervised the study and wrote the manuscript. All authors read and approved the final manuscript.

## Supplementary Material

Additional file 1 **Microarray analysis of *35S***_***pro***_***::FIL:GR *seedlings following dexamethasone treatment. **This file contains lists of genes that were identified as being differentially expressed in the shoot tissue of *35S*_*pro*_*::FIL:GR *lines exposed to DEX for 4 h or 8 h. Click here for file

Additional file 2 **FIL-response genes that are differentially expressed at 4 h and 8 h following DEX treatment or at 8 h following DEX treatment. **This file contains lists of genes that are have been placed into two groups. Group A genes are differentially expressed at 4 h and 8 h following DEX treatment, whereas Group B genes are differentially expressed at 8 h but not 4 h following DEX treatment. ( Click here for file

Additional file 3 **Histochemical staining of seedlings treated with DEX and CHX. **This figure shows the effectiveness of DEX and DEX/CHX treatments on control plants. Histochemical staining for GUS activity in ten-day-old *35S*_*pro*_^*I*^*> > GUS *seedlings exposed to a mock DEX/CHX treatment (A), CHX (B), DEX (C) and DEX/CHX (D) for 9 h. Scale bars are 1 mm. Click here for file

Additional file 4 **Vegetative phenotypes associated with steroid-induced activation of abaxially expressed *FIL*and constitutively expressed *YAB3*. **This figure shows the phenotype of *FIL*_*pro*_*::FIL:GR *plants and *35S*_*pro*_*::YAB3:GR *plants continuously exposed to DEX. (A) Fourteen-day-old *FIL*_*pro*_*::FIL:GR *plants grown on soil and sprayed with DEX (see Methods). (B,C) *35S*_*pro*_*::YAB3:GR *plant grown on media without DEX (B) or in the presence of DEX (C). Scale bars are 1 mm. Click here for file

Additional file 5 **Vegetative phenotype associated with continuous FIL activation in different mutants backgrounds.** This figure shows the DEX-inducible phenotype and leaf epidermal cell morphology of mutant plants harbouring the *35S*_*pro*_*::FIL:GR *construct. (A-D) Twenty-day-old *35S*_*pro*_*::FIL:GR *(A), *35S*_*pro*_*::FIL:GR*/*arf4* (B), *35S*_*pro*_*::FIL:GR*/*kan1* (C) and *35S*_*pro*_*::FIL:GR*/*arf4 kan1 *(D), plants grown on soil and sprayed with DEX (see Methods). (E-I) SEM of the adaxial surface of a leaf taken from *35S*_*pro*_*::FIL:GR *plants (E, F), a *35S*_*pro*_*::FIL:GR*/*arf4 *plant (G), a *35S*_*pro*_*::FIL:GR*/*kan1 *plant (H) or a *35S*_*pro*_*::FIL:GR*/*arf4 kan1 *plant (I). Plants received a mock treatment (E) or were sprayed with DEX (F-I). Scale bars are 2 mm (A-D) and 100 μM (E-I). Click here for file

Additional file 6 **Primer sequences used for qRT-PCR and cloning. **This file contains a list of all the oligonucleotides used for generating constructs and conducting qRT-PCR. Click here for file
