# Supplementary figures and images for: FILAMENTOUS FLOWER controls lateral organ development by acting as both an activator and a repressor
Source: BMC Plant Biol. 2012 Oct 1;12:176. doi: 10.1186/1471-2229-12-176 (PMC3520853; doi:10.1186/1471-2229-12-176)

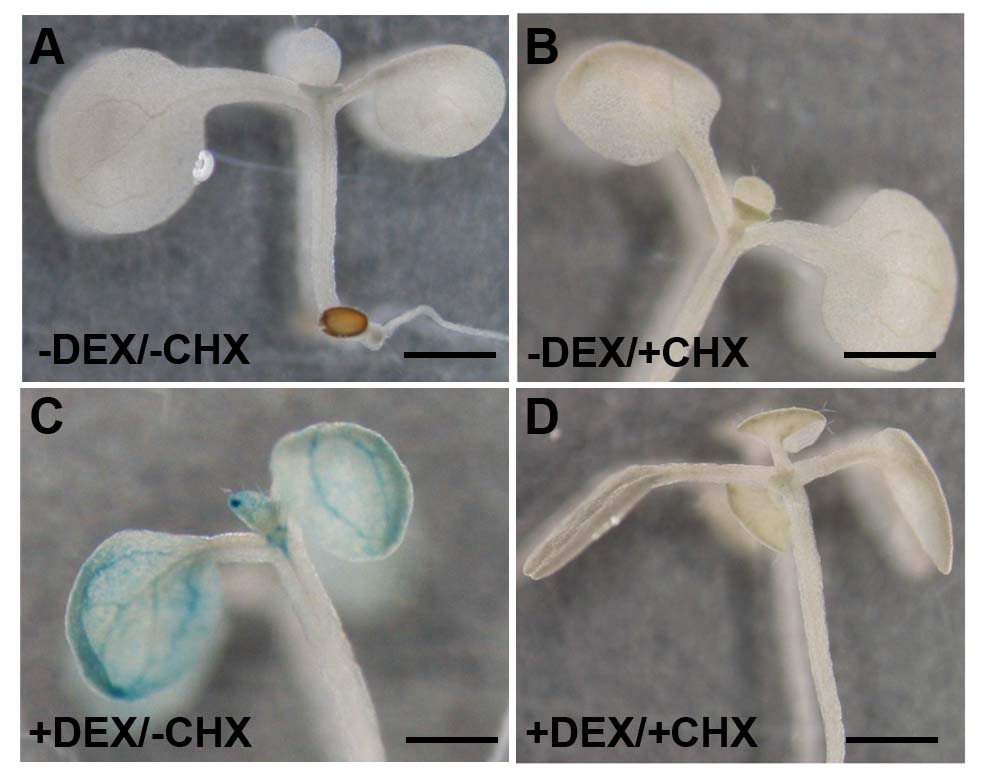

Supplement: Additional file 3 — Histochemical staining of seedlings treated with DEX and CHX. This figure shows the effectiveness of DEX and DEX/CHX treatments on control plants. Histochemical staining for GUS activity in ten-day-old 35SproI> > GUS seedlings exposed to a mock DEX/CHX treatment (A), CHX (B), DEX (C) and DEX/CHX (D) for 9 h. Scale bars are 1 mm. [file 1471-2229-12-176-S3.jpeg]

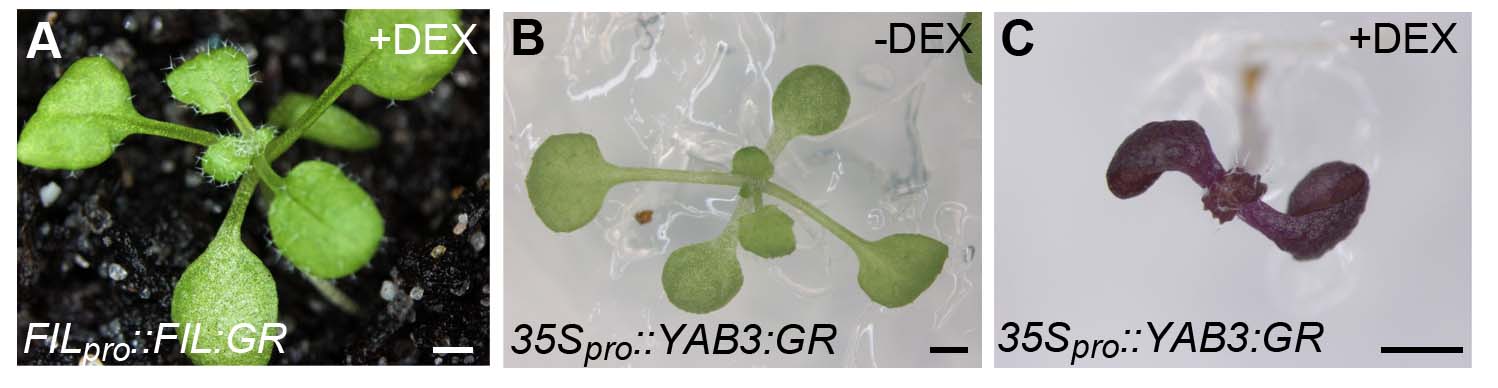

Supplement: Additional file 4 — Vegetative phenotypes associated with steroid-induced activation of abaxially expressed FILand constitutively expressed YAB3. This figure shows the phenotype of FILpro::FIL:GR plants and 35Spro::YAB3:GR plants continuously exposed to DEX. (A) Fourteen-day-old FILpro::FIL:GR plants grown on soil and sprayed with DEX (see Methods). (B,C) 35Spro::YAB3:GR plant grown on media without DEX (B) or in the presence of DEX (C). Scale bars are 1 mm. [file 1471-2229-12-176-S4.jpeg]

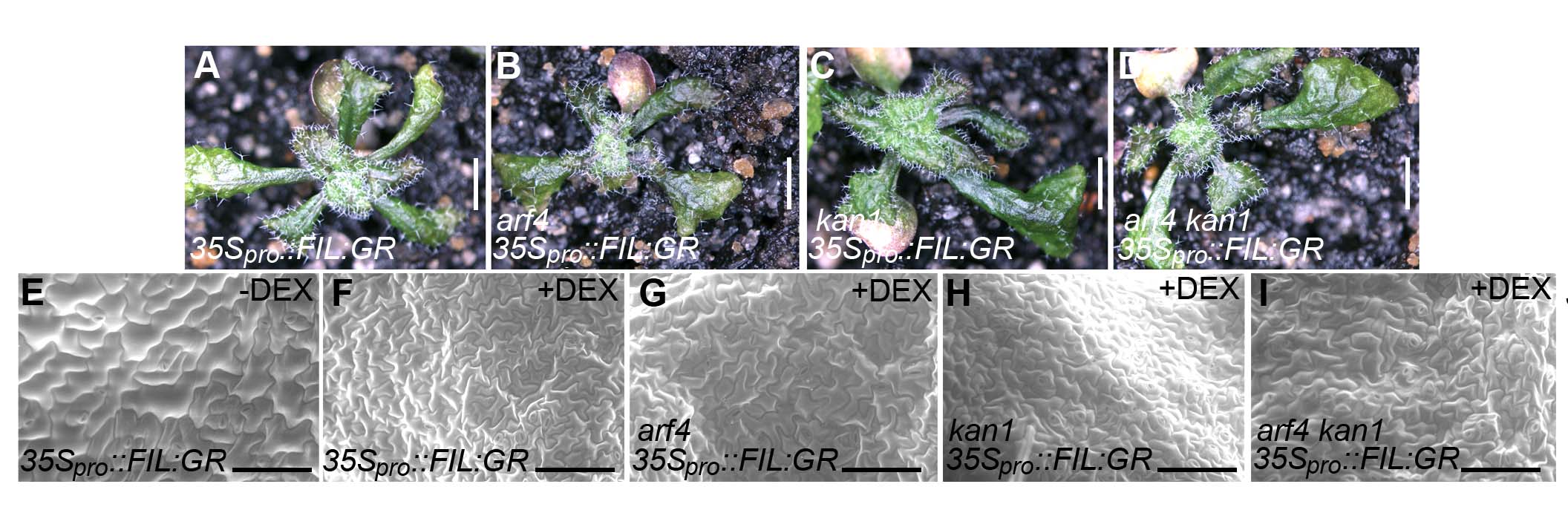

Supplement: Additional file 5 — Vegetative phenotype associated with continuous FIL activation in different mutants backgrounds. This figure shows the DEX-inducible phenotype and leaf epidermal cell morphology of mutant plants harbouring the 35Spro::FIL:GR construct. (A-D) Twenty-day-old 35Spro::FIL:GR (A), 35Spro::FIL:GR/arf4 (B), 35Spro::FIL:GR/kan1 (C) and 35Spro::FIL:GR/arf4 kan1 (D), plants grown on soil and sprayed with DEX (see Methods). (E-I) SEM of the adaxial surface of a leaf taken from 35Spro::FIL:GR plants (E, F), a 35Spro::FIL:GR/arf4 plant (G), a 35Spro::FIL:GR/kan1 plant (H) or a 35Spro::FIL:GR/arf4 kan1 plant (I). Plants received a mock treatment (E) or were sprayed with DEX (F-I). Scale bars are 2 mm (A-D) and 100 μM (E-I). [file 1471-2229-12-176-S5.jpeg]
