# Supplementary material for: FILAMENTOUS FLOWER controls lateral organ development by acting as both an activator and a repressor
Source: BMC Plant Biol. 2012 Oct 1;12:176. doi: 10.1186/1471-2229-12-176 (PMC3520853; doi:10.1186/1471-2229-12-176)
Supplement: Additional file 6 — Primer sequences used for qRT-PCR and cloning. This file contains a list of all the oligonucleotides used for generating constructs and conducting qRT-PCR. [file 1471-2229-12-176-S6.docx]

| **Additional file 6.** Primer sequences used for qRT-PCR and cloning. | |
| --- | --- |
| Name | Sequence |
| qRT-PCR oligonucleotides | |
| qCZF2-F | 5’-gtgacaaggcgttttcgtct-3’ |
| qCZF2-R | 5’-agcttttccggtgacttgc-3’ |
| qWRKY33-F | 5’-gggaaacccaaatccaaga-3’ |
| qWRKY33-R | 5’-gtttcccttcgtaggttgtga-3’ |
| qPAP1-F | 5’-aaatggcaccaagttcctgt-3’ |
| qPAP1-R | 5’-tcagagctaagttttcctctcttgat-3’ |
| qERF59-2-F | 5’-agagtgtggcttgggacatt-3’ |
| qERF59-2R | 5’-aagccgcctgatcataagc-3’ |
| MATE-QF1 | 5’-AGCGGTTAGCCACGGTTAT-3’ |
| MATE-QR1 | 5’-CCCACATATCAGACCGATCC-3’ |
| ETT-QF1 | 5’-ATCTCCAATTCAGGCAGCTT-3’ |
| ETT-QR1 | 5’-AAAGTCTGTGGCGCGAATC-3’ |
| ARF4-QF2 | 5’-TTAATCCCCAAATCGTCTGC-3’ |
| ARF4-QR1 | 5’-TCTTGAAGTGGAAGCAGAGTG-3’ |
| UBC17-QF1 | 5’-TCTCAATGCTCTCCAGCTCA-3’ |
| UBC17-QR1 | 5’-TGGAAACGCCACCTAGTTTC-3’ |
| qKAN1-F | 5’-ctaacaagcctgctgcttca-3’ |
| qKAN1-R | 5’-cgtttccatttatgcccatt-3’ |
| KAN2-QF1 | 5’-CTCAGACGACTGGATGTTCG-3’ |
| KAN2-QR1 | 5’- GCTTCTCCGGAAGAATTGGT-3’ |
| KAN3-QF1 | 5’-GAGGAGTAAGGGCTCCAAGG-3’ |
| KAN3-QR1 | 5’- ATTTTGGGGTTGCTCTTTCA-3’ |
| MPK3-QF1 | 5’-TGGCCATTGATCTTGTTGAC-3’ |
| MPK3-QR2 | 5’-ATTCGGGTCGTGCAATTTAG-3’ |
| ABC-QF1 | 5’-GGAGGAGATGCTACTGAAACC-3’ |
| ABC-QR1 | 5’-AACTCCTCTTTCACCCACCA-3’ |
| qARF10-F | 5’-ttcttctagaatcagttggtttatgg-3’ |
| qARF10-R | 5’-cggttcgtcccaagctac-3’ |
| NAC1-QF1 | 5’-CCTCCCAATCATTCTCTGAGC-3’ |
| NAC1-QR1 | 5’-CAAAACAGCTTCCCATGTTG-3’ |
| Exp Prot-QF1 | 5’-GGGCTTGGAGAATCTTGTTT-3’ |
| Exp Prot-QR1 | 5’-GGTCATCGAAAATGCATGAG-3’ |
| KIN2-QF1 | 5’-CATTTGTTTGGGCCTTGACT-3’ |
| KIN2-QR1 | 5’-TCCAGCAAACCTAGGGAAAG-3’ |
| SINA-QF1 | 5’-GCTCGATTTTCCGGTTCATA-3’ |
| SINA-QR1 | 5’-TGACATCACACTTGGCTCCT-3’ |
| KIN1-QF1 | 5’-TGGGGATTGCATTACCCTTAT-3’ |
| KIN1-QR1 | 5’-CGATGACCACTTGCACAATC-3’ |
| ERF5-QF1 | 5’-GAAGAAAAGCCGTGCTCAAC-3’ |
| ERF5-QR1 | 5’-GAAGCTCTTCATGCACATCAC-3’ |
| AS1-QF1 | 5’-TCAAGAGCAACTTCCATCACA-3’ |
| AS1-QR1 | 5’-GCCTCTCTCACTTTGGTCTTT-3’ |
| qNPH3-1F | 5’-ttgtaccgagccattgatacat-3’ |
| qNPH3-1R | 5’-attctgagccgcgtgaag-3’ |
| qIAA17-F | 5’-tttgtccaacatgttcagctct-3’ |
| qIAA17-R | 5’-ccaaatccatcaatttcctctc-3’ |
| EXP8-QF1 | 5’-GCCAGCTTTTCTTCAGATCG-3’ |
| EXP8-QR1 | 5’-TGTCCGTTGATCGTAAACCT-3’ |
| CPD-QF1 | 5’-GGCTAGGGTTGCACTCTCTG-3’ |
| CPD-QR1 | 5’-GGTACCGTTTCTGCGTTCTT-3’ |
| EXP11-QF1 | 5’-GGAAGTGACGCTTCTGGAAC-3’ |
| EXP11-QR1 | 5’-GAACAGAGCCGTGCTTAACG-3’ |
| TUB7-F | 5’-CATTTGCTTCGGTACACTCCA-3’ |
| TUB7-R | 5’- CCAGGGAACCTAAGACAGCA-3’ |
| ACT2-F | 5’-TCTTCCGCTCTTTCTTTCCA-3’ |
| ACT2-R | 5’-TCCTTCTGGTTCATCCCAAC-3’ |
| TCTP-F | 5’-ACACCCAAGCTCAGCGAAGAA-3’ |
| TCTP-R | 5’-CATGCATACCCTCCCCAACAA-3’ |
| Oligonucleotides for yeast constructs | |
| Gram-Eco-F | GGAATTCATGTCCTCTTCATCTGCT |
| Gram-Eco-R | GGAATTCTTAATAGGGAGACACACC |
| Prol-Eco-F | GGAATTCATGGATGTACTTGAGCAA |
| Prol-Eco-R | GGAATTCTTAAATAAAGTACCCCTT |
| AmYAB2-Eco-F | GGAATTCATGTCACTGGATATGACC |
| AmYAB2-Eco-R | GGAATTCTTAGTAGAATCCAAGAGA |
|  |  |
| Oligonucleotides for plant transformation constructs | |
| FIL-F3 | 5’-GCTCTAGACCCCTTCTTACAAAAAAG-3’ |
| FIL-R1.1 | 5’-ATGGATCCACACCAACGTTAGCAGC-3’ |
| cFIL-Kpn | 5’-GGGGTACCCCTTCTTACAAAAAAGATG-3’ |
| Fil R-X | 5’-CCTCTAGATTAATAAGGAGTCAC-3’ |
| FIL-TRX | 5’- tttctagattcatgggactctcttgtcttt-3’ |
| FIL-TRB | 5’- tttggatccGGGACTCTCTGTCTTTTC-3’ |
| pFIL-Xho | 5’-ccgCtCgagtttaacgagtgaagcaga-3’ |
| pFIL-KpnI | 5’-GGGGTACCGAAGGGGAAAAATATTGG-3’ |
|  |  |
| Oligonucleotides for transactivation constructs | |
| FIL-TA-F | 5’-AGATCTGCTGCTGCTGCTAGATCCATGTCTATGTCGTC  TATGTCC-3’ |
| FIL-TA-R | 5’-GGATTCCTTAATAAGGAGTCACACC-3’ |
| FIL:SRDX-R | 5’-CTAGATCAAGCAAAACCGAGCCTGAGTTCGAGATCGAG  ATCGAGAG-3’ |
| YAB3-TA-F | 5’-AGATCTGCTGCTGCTGCTAGATCCATGTCGAGCATGTC  CATGTC-3’ |
| YAB3-TA-R | 5’-AGATCTCTAGTTATGGGCCACCCC-3’ |
| YAB2-TA-F | 5’-AGATCTGCTGCTGCTGCTAGATCCatgtctgtagattt  ctcatc-3’ |
| YAB2-R1 | 5’-CCTTAGTAATAGCCATTAGAC-3’ |
| YAB5-TA-F | 5’-AGATCTGCTGCTGCTGCTAGATCCATGGCTAACTCTGT  GATGGC-3’ |
| YAB5-R1 | 5’-CCTTAGGCTATCTTAGCTTGCTTG-3’ |
|  |  |
